# Phage Therapy as an Alternative Treatment Modality for Resistant *Staphylococcus aureus* Infections

**DOI:** 10.3390/antibiotics12020286

**Published:** 2023-02-01

**Authors:** Salman Sahab Atshan, Rukman Awang Hamat, Musheer A. Aljaberi, Jung-Sheng Chen, Shih-Wei Huang, Chung-Ying Lin, Benjamin J. Mullins, Anthony Kicic

**Affiliations:** 1 Department of Medical Science, Collage of Dentistry, University of Basrah, Basrah 61004, Iraq; 2 School of Population Health, Curtin University, Perth, WA 6152, Australia; 3 Wal-yan Respiratory Research Centre, Telethon Kids Institute, The University of Western Australia, Perth, WA 6009, Australia; 4 Department of Medical Microbiology, Faculty of Medicine and Health Sciences, Universiti Putra Malaysia, Serdang 43400, Selangor, Malaysia; 5 Faculty of Medicine and Health Sciences, Taiz University, Taiz 6803, Yemen; 6 Department of Medical Research, E-Da Hospital, I-Shou University, Kaohsiung 824, Taiwan; 7 Institute of Environmental Toxin and Emerging Contaminant, Cheng Shiu University, Kaohsiung 833, Taiwan; 8 Center for Environmental Toxin and Emerging-Contaminant Research, Cheng Shiu University, Kaohsiung 833, Taiwan; 9 Institute of Allied Health Sciences, College of Medicine, National Cheng Kung University, Tainan 701, Taiwan; 10 Department of Respiratory and Sleep Medicine, Perth Children’s Hospital, Nedlands, WA 6009, Australia; 11 Centre for Cell Therapy and Regenerative Medicine, School of Medicine and Pharmacology, The University of Western Australia, Perth, WA 6009, Australia

**Keywords:** phage therapy, antibiotics resistant, *S. aureus*, biofilms, infection

## Abstract

The production and use of antibiotics increased significantly after the Second World War due to their effectiveness against bacterial infections. However, bacterial resistance also emerged and has now become an important global issue. Those most in need are typically high-risk and include individuals who experience burns and other wounds, as well as those with pulmonary infections caused by antibiotic-resistant bacteria, such as *Pseudomonas aeruginosa*, *Acinetobacter sp,* and *Staphylococci*. With investment to develop new antibiotics waning, finding and developing alternative therapeutic strategies to tackle this issue is imperative. One option remerging in popularity is bacteriophage (phage) therapy. This review focuses on *Staphylococcus aureus* and how it has developed resistance to antibiotics. It also discusses the potential of phage therapy in this setting and its appropriateness in high-risk people, such as those with cystic fibrosis, where it typically forms a biofilm.

## 1. Introduction

Bacterial infections are the cause of major health problems, but after the advent of antibiotics, there was the view that the issue was resolved. However, one pathogen of current global importance, *Staphylococcus aureus* (*S. aureus*), has developed drug resistance mechanisms to the currently available antibiotics, including cloxacillin, vancomycin, daptomycin, and others, and is resultantly responsible for over two million infections and over 23,000 deaths in the United States alone each year [1,2,3]. With antibiotic development by the pharmaceutical industry waning, there are many unanswered questions, including what alternatives are being developed and whether bacteriophages could be a solution. 

Bacteriophages (phages) are found in all habitats, and their interaction with bacteria has attracted greater attention from scientists for almost a decade [4]. Bacteriophage therapy or phage therapy (PT) that consists of specific virulent bacteriophages can be used for targeting multidrug-resistant bacteria and can mimic the action of an antibacterial agent [5]. Indeed, PT has currently been used to treat patients with methicillin-resistant *Staphylococcus aureus* (MRSA) infections [6,7,8,9]. According to the NCBI database, a total of 69 genomes of virulent staphylophages have been deposited, with 26 and 43 genomes belonging to the family of *Podoviridae* and *Myoviridae* phages, respectively [10]. The high efficiency of staphylophages in the therapeutic cocktail is attributed to its broad host range and high lytic capabilities [11].

Although various successful investigations have explored using singular or multiple phage types (phage cocktails), a definitive answer on their safety and efficacy as part of standard clinical care is still unknown [12]. This review describes one of the challenges faced prior to its clinical translation, namely, the approaches utilized by *S. aureus* to develop resistance and form biofilms, and then discusses the potential of bacteriophage to treat these infections as part of a therapeutic treatment strategy. Several challenges to the current recommendation for treating *S. aureus*-specific infections are also highlighted.

## 2. Narrative Review

### 2.1. Staphylococci

Staphylococci are frequently isolated as the causal agent of bacterial infections in humans, and its management remains challenging due to the advent of multidrug-resistant strains [13]. It commonly causes surgical wound infections and pneumonia and is the second most common cause of blood infections [14]. Importantly, *S. aureus* has been extremely efficient at developing resistance to the most recent antibiotic classes. Although methicillin-resistant *S. aureus* (MRSA) was quite rare between the 1960s and 1980s, the problem escalated in the mid-1990s when particular ‘epidemic’ strains became established in hospitals throughout the UK, which were caused by SCC*mec* type 1. Several antibiotics targeting methicillin-resistant *S. aureus* (MRSA) have been developed and approved for use, including linezolid, daptomycin, tigecycline, ceftobiprole, telavancin, ceftaroline, dalbavancin, oritavancin, and tedizolid [15]. However, they have reduced efficacy when staphylococci establish as biofilms, typically seen with device-associated infections and endocarditis [16]. To evaluate the correlation and development of MRSA strains, research was conducted, leading to two postulated theories. The first suggested that all methicillin-resistant strains were descendants from the integration of SCC*mec* into the genome of *S. aureus* and with further mutations, namely, SCC*mec* types I–V evolving thereafter [17]. The second multi-clone theory, which is more universally accepted, implies that SCC*mec* integrated into various *S. aureus* strains over time [18].

The global impact of MRSA infections is now being felt due to the ease of its transmission between patients and hospital staff. This has been eloquently exemplified by Dickmann et al. [19], who showed a high prevalence of MRSA, consequently leading to ventilator-associated pneumonia, chronic wound infection, bloodstream infection (bacteremia), and sepsis. However, community-MRSA strains can be easily transmitted within hospitals and from hospitals transmitted into the community [20]. Furthermore, the emergence of vancomycin resistant *S. aureus* (VRSA) has increased the potential risk of patient transmission even further [21]. Since the development of antibiotic resistance by *S. aureus* occurs at the genetic level, its genetic structure and the evolution of existing resistance are discussed in more detail in the subsequent sections.

### 2.2. Genetic Structure of Staphylococcus aureus

**Figure 1:**
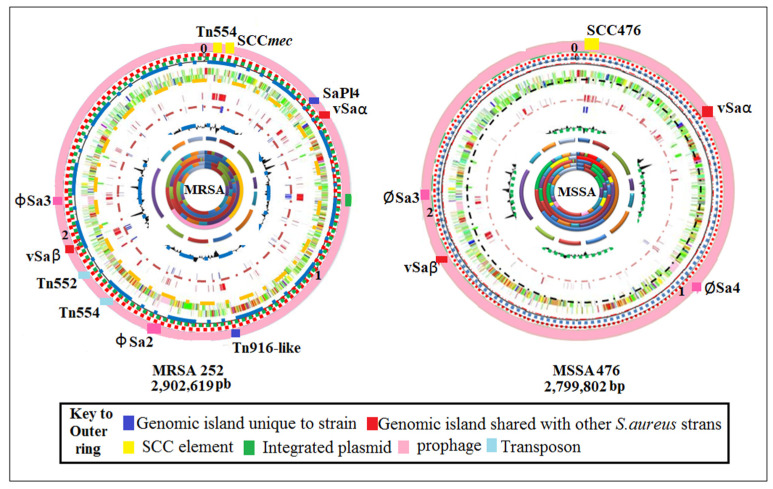
Circular diagram of the MRSA 252 and MSSA 476 chromosomes. Colored segments on the outer ring (pink): genomic islands and horizontally acquired DNA. Rings inside and moving internally represent annotated CDS (in Mbp). tRNA and rRNA (green), additional DNA compared to other *S. aureus* strains (red and blue), percentage of G + C content, and G + C deviation (>0%, olive; <0%, purple). Predicted function coding for CDSs: pathogenicity/adaptation (royal blue); energy metabolism (black); information transfer (red); surface-associated (dark green); degradation of large molecules (cyan); degradation of small molecules (magenta); central/intermediary metabolism/intermediary metabolism (multi-colored); unknown (light green); regulators (light blue); conserved hypothetical (multi-colored segments); pseudogenes (brown); phage plus insertion sequence elements (pink); miscellaneous (grey). Adapted from Holden et al. [27].

The *S. aureus* genome consists of a circular double-stranded chromosome as well as several plasmids. The number of complete and draft genomes has grown exponentially, with hundreds now available in the NCBI repository [22,23]. Upon examination, conservational similarities and differences are seen between methicillin-sensitive *S. aureus* (MSSA) and MRSA (Figure 1). For example, the genomic core (representing ~75% of the entire genome) is extremely conserved between both MRSA and MSSA isolates and contains genes associated with housekeeping and other metabolic functions [24]. In addition to the core genome, accessory genomes can be obtained from other bacterial species by lateral gene transfer [25]. This domain accounts for ~25% of the entire genome and consists of mobile genetic elements such as virulence factors, chromosome cassettes, genomic plasmids, transposons, and antibiotic resistance dynamics [26].

### 2.3. Staphylococcal Cassette Chromosome (SCC)

**Figure 2:**
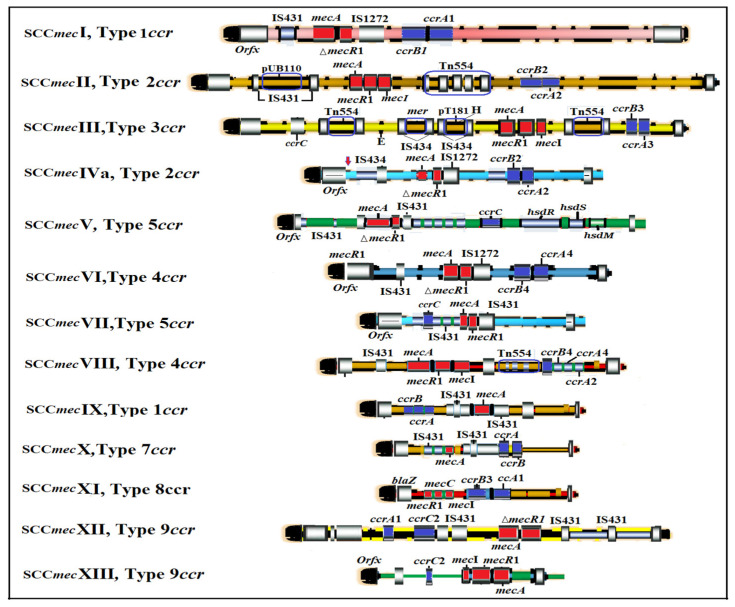
Scale representation and comparison of the 13 SCC*mec* types. *Mec* gene complex (red), *ccr* gene complex (indigo), ORFs within the SCC*mec* elements (blue), and chromosomal ORFs (black). (Adapted with permissions from Ref. [33]. 2009, American Society for Microbiology).

As an important basic mobile genetic element, the SCC serves as the motor vehicle for gene substitution among *Staphylococcus* species. It typically contains the *mec* gene (although not always present), which codes for methicillin resistance, regulatory genes, as well as the cassette chromosome recombinases (*ccr*), which enables the integration and cutting of SCC*mec* [28]. SCC*mec* typing enables structural determination of SCC*mec* and can assist in inferring the origins of particular MRSA strains [29]. Each SCC*mec* element integrates at the same site, which is a precise site at the 3‘ end of a unique ORF (open reading frame) of unknown function, designated orfX [30]. To date, 13 different types of SCC*mec* (I-XIII) have been defined according to their subparts, namely, the *ccr* and the *mec* complexes [31,32] (Figure 2).

### 2.4. Update on Treatment of S. aureus Infections and Its Associated Challenges

Treatment agents currently available for *S. aureus* infections include cloxacillin, flucloxacillin, cefazolin, ceftaroline, ceftobiprole, vancomycin, daptomycin, linezolid, teicoplanin, tigecycline, co-trimoxazole, doxycycline, clindamycin, telavancin, dalbavancin, oritavencin, and others [34]. Several antibiotic guidelines for treating *S. aureus* infections have been established and regularly updated according to the epidemiological data of antibiotic resistance patterns, national and global surveillance of resistance, and efficacy data of newly anti-staphylococcal agents [35]. The guideline consists of recommended antibiotics (weak and strong recommendations) and therapy regimes (dosage and duration) used for specific types of infections such as skin and soft-tissue infections (SSTIs), urinary tract infections (UTI), bone and joint infections, bacteremia, endocarditis, pneumonia, and meningitis [36]. However, infections caused by MRSA deserve special attention as common antibiotics used for MSSA are no longer effective against them, and their propensity to rapidly spread, causing an outbreak, is very common [37,38]. For example, abscesses are generally managed by performing incision and drainage only; however, antibiotics must be given together with this surgical procedure for an abscess caused by MRSA PFGE strain type USA300 in the United States [39,40]. Nonetheless, different countries may have their own policies regarding MRSA treatment strategies. For example, antibiotic therapy after drainage is not recommended even for uncomplicated abscesses caused by USA300 strains due to its low prevalence in the United Kingdom (UK) [41].

Topical antiseptics such as hydrogen peroxide 1% cream remain an effective option for patients with uncomplicated impetigo due to MRSA [36]. Despite new anti-MRSA agents that have been officially licensed, there is no solid evidence to establish the optimal agent(s) for specific infections caused by MRSA. Retapamulin ointment 1%, an agent of the pleuromutilin class, is still inferior to oral linezolid in a clinical trial, thus, limiting its use for this condition [42]. Ceftobiprole, dalbavancin, and tedizolid are not recommended for the SSTIs caused by MRSA due to inconcrete evidence from the clinical trials [43,44,45]. In fact, major side effects, including thrombocytopenia and optic neuropathy, have been reported in patients treated with linezolid and tedizolid [46].

Limited data from clinical trials also suggested that complicated (non-catheter related) UTIs caused by MRSA should be treated with intravenous glycopeptides such as vancomycin or teicoplanin [36]. Similarly, intravenous glycopeptides are strongly recommended for treating necrotizing pneumonia due to Panton–Valentine leucocidin (PVL)-producing MRSA strains despite no recent update about the existing guideline in the UK since 2008 [47]. As for hospital-acquired pneumonia (HAP) caused by MRSA, a similar approach to therapy is not highly recommended due to a lack of data based on clinical trials [36]. On another note, serious adverse events such as multiorgan failure and septic shock have been reported in patients with pneumonia receiving telavancin compared with those receiving vancomycin [48]. In addition, the inhibitory interaction of daptomycin with lung surfactant has been observed; hence, it is not licensed as an anti-MRSA agent for pneumonia [49]. Recently, a cross-resistance towards daptomycin has been reported among vancomycin-resistant *S. aureus* isolates, which is caused by a mutation in the *mprF* gene [50]; thus, stringent infection control measures should be advocated.

Rifampicin is used as an adjunct therapy in patients with MRSA bone or joint infections for its ability to penetrate the biofilm [47]. There has been an increasing preference for using dalbavancin among clinicians for its long half-life and convenient dosing regimen [51]. Nonetheless, recent evidence from the clinical trials fails to support the use of these drugs for this condition. Instead, intravenous glycopeptides are highly recommended with proper therapeutic drug monitoring in view of the nephrotoxic effect associated with the prolonged use of vancomycin [36,52]. A similar recommendation is proposed for MRSA bacteremia except for the use of teicoplanin. Linezolid, telavancin, and ceftaroline can be used as alternative second-line drugs if vancomycin is contraindicated [53,54], although it is not highly recommended by the recent guidelines [36]. In meningitis cases, intravenous vancomycin is highly recommended, and rifampicin may be considered for severe infection [36]. Intrathecal vancomycin can be performed directly into the ventricles of the brain if vancomycin is not effective via the intravenous route [36]. However, a neurotoxic effect of vancomycin has been observed at the cellular level following an intrathecal administration of the drug *in vivo*; hence, the intrathecal dose needs to be properly regulated [55]. Bacteriostatic drugs for treating meningitis, such as chloramphenicol, clindamycin, or linezolid, are strongly prohibited [36]. Vancomycin has also been recommended for the treatment of native or prosthetic valve MRSA endocarditis [56]. As an alternative, a combination of daptomycin and another anti-MRSA agent is recommended [36].

It seems that vancomycin is highly recommended for the majority of MRSA-specific infections. Indeed, vancomycin or daptomycin, in combination with other anti-MRSA agents, have effectively been used for treating invasive MRSA infections [57]. However, their effectiveness is diminishing due to increasing resistance amongst MRSA strains, compounded further by the fact that many have existing resistance to several other classes of antibiotics [58,59]. For example, cross-resistance towards daptomycin has been reported among vancomycin-resistant *S. aureus* isolates, which is caused by a mutation in the *mprF* gene [50]. Although numerous anti-staphylococcal antibiotics have been developed during the past 30 years, and several guidelines have been revisited, infection with *S. aureus* continues to result in severe morbidity and, sadly, mortality, creating an increasingly urgent need for more appropriate treatments and better patient management. Apart from the adverse events caused by several anti-MRSA agents and inconcrete findings from the most available clinical trial data, the development of antibiotic resistance and biofilm formation by *S. aureus* has created a new platform for us to look for other alternative therapies, such as phage therapy. This is further elaborated in the respective sections below.

### 2.5. Resistance to Antimicrobial Therapy

A pathogen’s success in causing infection depends on two factors, (i) developing resistance to antibiotics and (ii) virulence factors. Resistance in *S. aureus* is obtained via the acquisition of mobile genetic elements (SCC*mec*) containing resistance genes such as *mec*A encoding a unique protein called penicillin-binding protein 2a (PBP2a). Its low affinity for β-lactam antibiotics thus enables it to substitute the biosynthetic functions of PBPs [60]. In addition to SCC*mec*, other mobile genetic elements involved in resistance acquisition include plasmids, transposons, insertion sequences, integrons, integrative-conjugative elements, and pathogenicity islands [61]. One staphylococcal conjugative plasmid (pGO1/pSK41) has been directly shown to have contributed to resistance to aminoglycosides, penicillin, trimethoprim, bleomycin, tetracycline, macrolides, lincosamide, streptogramin B, and more recently linezolid and vancomycin [62]. Furthermore, transposons, which themselves are a group of mobile genetic elements, can transfer resistance genes due to their ability to transfer between plasmids or from a DNA chromosome to a plasmid (or vice versa) [63]. Thus, antibiotic resistance emerges rapidly through the acquisition of antibiotic-resistance genes from other strains of *S. aureus* or even from other genera. The development of resistance due to antibiotic treatment failure results in outbreaks of multiple drug-resistant (MDR) strains, typically in hospital institutions as well as the general population [64]. For example, MRSA infections in US hospitals have almost doubled, more than any other nosocomial pathogens [65]. Indeed, a significant proportion of all health care-associated *S. aureus* infections appear to be caused by MRSA [66]. This observation has been further corroborated by Ehsanollah et al., who showed that MRSA is the most important nosocomial pathogen in Malaysian hospitals, rising from 10% in 1985 to 44.1% in 2007 [67]. *S. aureus* was first observed with intermediate susceptibility to vancomycin in Japan in 1997 [68]. However, four clinical isolates of *S. aureus*, derived from the USA, were subsequently found to be vancomycin-resistant [69]. These observations suggest that *S. aureus* infections continue to be challenging to treat as new clones of MRSA emerge from hospitals and from the community. Early treatment and proper monitoring are crucial steps in the prevention of MRSA infections [70], however, are hindered further when they acquire resistance genes from the same and/or different genera of bacteria. The story of antibiotic resistance begins with penicillin, produced by the mold *Penicillium rubens* [71]. Affecting the production of peptidoglycan, which is essential for cell wall synthesis [72], it was first reported to successfully treat infections in humans in 1940 by Chain and colleagues [73]. Shortly after, Kirby and colleagues published the first description of penicillin resistance [74], caused by a plasmid-encoded penicillinase that resultantly hydrolyses the beta-lactam ring [75]. Although initially rare, most penicillinase-producing *S. aureus* strains isolated from hospitalized patients are currently resistant to penicillin [76]. Subsequent to penicillin, a new variant called methicillin was introduced in 1961 [77], but resistance quickly established [78] and resulted from the acquisition of *mecA* [79]. Interestingly, SCC*mec* was not identified in *S. aureus* before this, and thus it has been postulated that it originated from *Staphylococcus sciuri* since it carries a homologous gene for *mec*A. Of significance, when this homolog gene (*pbp*D) has been introduced into MSSA strains, it confers to an MRSA phenotype [80].

In clinical settings, methicillin was replaced by oxacillin due to its acid stability; however, oxacillin-resistant *S. aureus* strains (ORSA) subsequently developed [81]. Vancomycin, a glycopeptide antibiotic, was then introduced in 1956 and worked effectively for more than 40 years before so-called vancomycin-intermediated *S. aureus* (VISA) were observed [82]. *S. aureus* strains present with resistance to vancomycin were then observed, including; low-level-resistant vancomycin-resistant *S. aureus* (LLR-VRSA) and high-level-resistant vancomycin-resistant *S. aureus* (HLR-VRSA) [83]. Eleven VRSA strains were identified and reported in 2009 in three geographically distinct countries [84], and their resistance to vancomycin was caused by the *vanA* operon. Carried on a particular transposon, it is suggested to have inserted itself initially from an *Enterococcus* plasmid to a secondary endogenous plasmid in *S. aureus* [49]. Indeed, enterococci possess many mobile genetic elements, including transposons, which can be transferred to the same or different species of bacteria via horizontal gene transfer [85]. This purposeful breach in the *S. aureus* restriction barrier infers a new defense mechanism utilized by this bacterium to maintain the adaptive advantage, and the transmission of resistance genes will be further enhanced if such process occurs within the restricted space such biofilms. As the pipeline to develop new antibiotics wanes and resistance in different bacterial genes emerges, our ability to control bacterial infections in the near future will be greatly challenged [86].

### 2.6. Role of Biofilm Formation in S. aureus Infections

**Figure 3:**
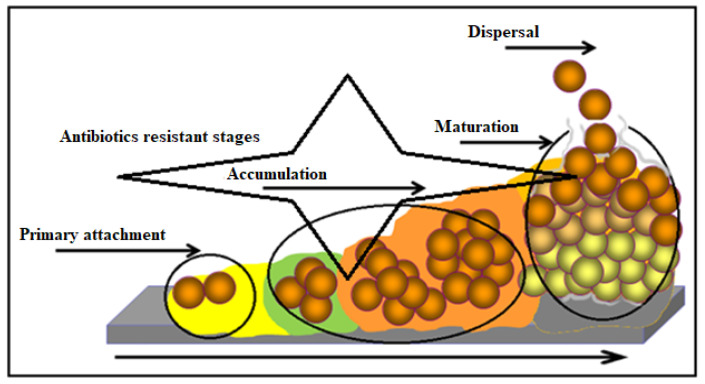
Model of biofilm progression showing distinct phases and microcolony structure. (Monroe [87].)

Microbial populations tend to adhere to surfaces by producing extracellular polymeric substances (EPS) and forming biofilms (Figure 3) [87]. Bacterial cells growing in a biofilm are difficult to treat by antibiotics since the EPS matrix prevents them from reaching the bacteria via physical repulsion or limited diffusion [88]. It has been estimated that of all bacterial infections, ~80% involve biofilm formation [89], and these appear to be 10–1000-fold more resistant to antibiotics compared to their planktonic counterparts [90]. The challenge to eradicate bacterial biofilms via antibiotics is well documented, with evidence suggesting some variabilities between drugs. For example, oxacillin, cefotaxime, and vancomycin have shown limited penetration, whereas amikacin and ciprofloxacin are far more effective in this function [91]. However, eradication of biofilms is very challenging, especially in clinical settings, as high rates of recurrent or chronic bacterial infections have been observed due to biofilms [92]. The development of biofilm-related *S. aureus* infections is always associated with medical devices or procedures used as part of patients’ management, such as indwelling catheters or implants, and chronic conditions such as chronic lung infections, chronic wounds, endocarditis, and others [92]. Several in vitro studies have demonstrated the promising role of bacteriophages in controlling *S. aureus* biofilms [93,94,95]. In addition, data on the complete resolution of *S. aureus* biofilm-related infections have been published recently by Pires et al. [96]. However, over time, bacterial biofilms have become increasingly resistant to yet more antibiotics, and innate host defense mechanisms, including antimicrobial peptides (AMPs) and neutrophil phagocytosis, have proven ineffective [97]. Moreover, the use of sub-minimal inhibitory concentration (MIC) of daptomycin and tigecycline could activate a signal for the induction of virulence genes in *S. aureus* via the regulation of biofilm adhesion factor genes and its exoproteins [98].

Collectively, as described in this review, staphylococci readily form antibiotic resistance, whether in free planktonic forms or biofilms. It can adapt over very short periods when a new antibiotic is introduced to develop resistance, which ultimately increases these infections’ economic and healthcare burden. Thus, an alternative treatment regimen that permanently eliminates infection is desperately required. One postulated solution is bacteriophage therapy.

### 2.7. Phage Therapy

**Figure 4:**
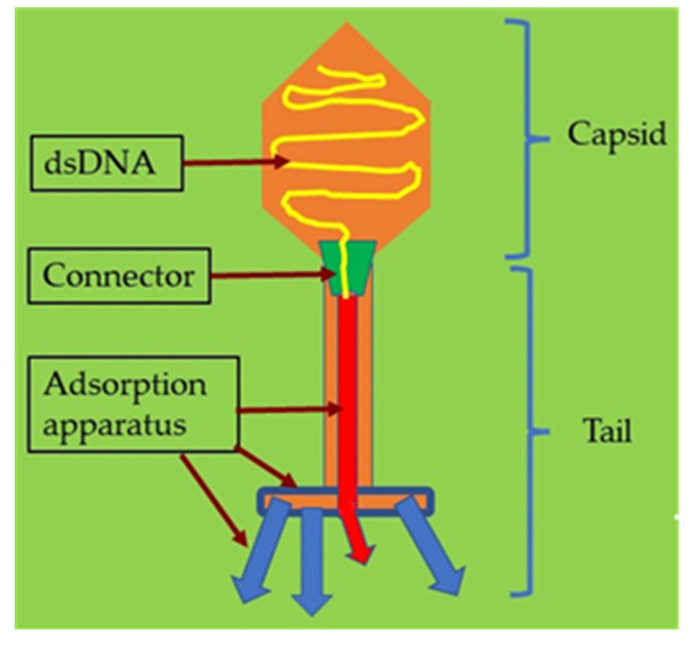
Illustration of a bacteriophage with its structure and components.

The term “phage” is defined as a type of virus that can enter and destroy bacterial cells whose potential was appreciated early in history [99,100]. The morphological structure of a bacteriophage is unique as it consists of a head that is filled with DNA or RNA and a tail that is used for the introduction of the genome into the bacterial cells (Figure 4). It has limited receptors on eukaryotic cells; hence, it may not enter mammalian cells, making it the most promising alternative therapeutic option in humans [101,102].

Their bactericidal activity begins with the binding of the virion to specific bacterial cell-surface receptors via phage receptor binding proteins (RBP), followed by the adsorption of phage DNA into a bacterium. This then initiates various molecular mechanisms which promote phage propagation and resultant bactericidal activity [103]. Bacteriophage therapy has been actively implemented since the early twentieth century after its discovery by Twort [104] and D’Hérelle [105]. Since this period, its popularity diminished as antibiotic use became the preferred method of treating infections in western countries. However, with the advent of antibiotic resistance, several recent investigations have explored the efficacy of treating bacterial infections with phages in the human setting [106,107,108]. Chan and Abedon first suggested that treatment strategies could be categorized by the number of phage types used, with ‘monophage therapy’ exploiting a single phage type and ‘polyphage therapy’ involving multiple phage types [109]. Levin and Bull initially emphasized the importance of accurately matching pathogens with specific phages. Although usually assessed in vitro, phage activity in this setting is not always predictive of their efficacy in vivo [110]. It has also been identified that phage preparations should be purified by filtration and passed through specific columns or resins to remove endotoxins from the phage lysate to a level appropriate for use, and toxicity testing of formulations in vivo should be conducted [111,112,113].

**Figure 5:**
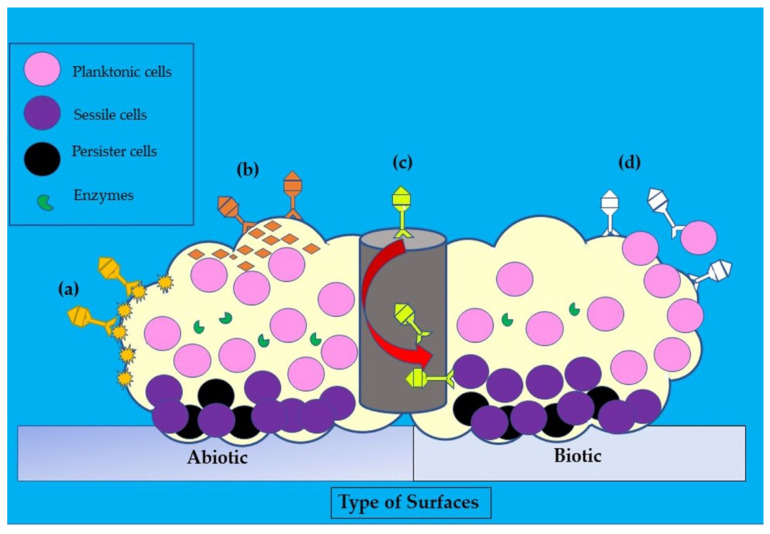
Illustration of the disruptive mechanisms of bacteriophages in *Staphylococcus aureus* biofilms on abiotic and biotic surfaces by the (**a**) expression of polysaccharide depolymerases which degrade extracellular matrix, (**b**) induction of host cells to produce exopolysaccharides (EPS)-degrading enzymes, (**c**) diffusion via biofilm water channels for inner layer penetration, and (**d**) adsorption to bacteria for biofilm penetration. The role of each bacteriophage is depicted in various colors (dark yellow, orange, yellow, and white).

As natural predators of bacteria, bacteriophages have several innate mechanisms to destroy biofilms (Figure 5). For example, degradation of the extracellular matrix is initiated by the induction of bacterial cells to produce exopolysaccharides (EPS)-degrading enzymes, which later break down the polysaccharides and proteins of the extracellular matrix (biofilms). Hence, this could facilitate the penetration of bacteriophages into the biofilms and later replicate in the bacterial cells and destroy them [114]. The phage can also express polysaccharide depolymerases, an enzyme that is able to degrade biofilms, as well as polysaccharide-forming capsules and lipopolysaccharides [115,116]. Interestingly, bacteriophages can enter the bacterial genome and integrate themselves, leading to “floating bacterial cells” that could interfere with the formation of biofilms [115].

Most phage/antibiotic studies have focused on *P. aeruginosa*, due to its significant clinical impact as an opportunistic pathogen, in cystic fibrosis (CF), burns, pneumonia, and urinary tract infections [117]. In one study conducted by Wright et al., utilizing a formulation of six phages at 10^5^ PFU direct in the ear as a treatment strategy for chronic otitis media caused by *P. aeruginosa*, patients were observed for up to 42 days’ post treatment, and encouragingly, there was a reduction in infection by over 50% [118]. Similarly, Gu et al. formulated a mixture of three phages to treat mice that were inoculated with *Klebsiella pneumoniae*. Results showed that a single intraperitoneal dose provided 1 h post-bacterial inoculation resulted in 100% recovery [119]. Another study by Chaudhry and colleagues treated biofilm-producing *P. aeruginosa* with two phages together or in combination with three antibiotics (ceftazidime, ciprofloxacin, and tobramycin). Significantly, only moderate activity was observed when each phage/antibiotic treatment was conducted singularly. However, synergistic activity was observed when phages and antibiotics were applied concurrently [120]. These findings importantly indicate that combining phages and antibiotics can be superior over the use of single agents to enhance the suppression of bacteria, enable more effective biofilm penetration, and prevent the emergence of phage resistance [121]. Trend et al. also screened multiple phages of *P. aeruginosa* (E79, F116, and P5) for activity against *P. aeruginosa* strains isolated from children with CF. Excitingly, the authors identified E79 as a possible new therapeutic phage candidate for *P. aeruginosa* lung infections in CF due to its broad antibacterial activity (91% of the tested strains were sensitive) [122].

Although much attention has focused on treating Gram-negative bacteria with phage, Estrella and colleagues demonstrated that many strains of staphylococci carried bacteriophages. These phages were able to lyse some but not all strains. However, by exposing staphylococci to several phages, a susceptibility pattern could be recognized, and similarities or differences between strains could be determined [123]. Since treatment options for MRSA are restricted, novel preventive strategies are currently being formulated, including phage/antibiotic combinations (e.g., vancomycin, daptomycin, and linezolid) [54,124,125]. Moreover, biofilm-forming *S. aureus* are well known for their resistance to high concentrations of antibiotics [126,127]. 

Lehman et al. were one of the first to characterize an *S. aureus* phage (AB-SA01) in detail. *S. aureus* phage (AB-SA01) is a bacteriophage cocktail produced by AmpliPhi Biosciences Corporation that consists of three naturally occurring, obligately lytic myoviruses related to *Staphylococcus* phage K belonging to the *Herelleviridae* family. Importantly, it did not contain any bacterial virulence or antibiotic resistance genes when sequenced. In addition, they confirmed that this phage’s inherent characteristics met the human use criteria and was predicted to remain active against circulating multidrug-resistant *S. aureus* strains for long enough to be useful under good manufacturing practices. Overall, results offered great promise, with AB-SA01 killing ~95% of *S. aureus* isolates [128]. Furthermore, there has been a rationale for specific formulations of the phages that make up AB-SA01 (phages Sa83, Sa87, and J-Sa36) where their synergistic activities to kill otherwise non-susceptible *S. aureus* strains have been shown [128]. Another study by Sanjay et al. used both a single and cocktail formulation comprising two phages (MR 5 and MR 10) to treat rats infected with MRSA [86]. Importantly, they showed effective treatment and better persistence stability when using a cocktail formulation of phage rather than when used singularly [129]. Using a lethal model of *S. aureus* ventilator-associated pneumonia (VAP), Prazack and colleagues demonstrated reduced mortality in mice treated with and without teicoplanin compared to the placebo [130]. Rahman and colleagues studied the synergistic effects between phage and antibiotics in biofilm-producing *S. aureus* strains [131]. Specifically, they used phage SAP-26 concurrently with azithromycin, vancomycin, or rifampicin and found a synergistic effect with SAP- 26 and rifampicin, where there was a 65% reduction in live cells. A similar significant bactericidal effect was observed when azithromycin or vancomycin was formulated with phage (40% and 60%, respectively). Importantly, this was the first study to illustrate the synergistic effects of a phage/antibiotic mixture against *S. aureus* biofilms [131]. Although it has been postulated that phages can degrade biofilm matrix using depolymerizes [93], they cannot be identified in many phage genomes to explain this activity [132]. Nevertheless, phages can replicate deep within a biofilm, disrupting its matrix. Following this interruption, the subsequent addition of antibiotics results in enhanced bacterial reduction [93].

Kumaran et al. also studied the synergistic activity of phage/antibiotic treatment against biofilm-forming *S. aureus* using the SATA-8505 phage with vancomycin, cefazolin, linezolid, dicloxacillin, and tetracycline, simultaneously or sequentially. Results demonstrated minimal bactericidal activity when both phage and antibiotic were concurrently added, which was significantly increased when phage treatment preceded antibiotics, particularly with vancomycin and cefazolin [133]. Subsequent studies by Akturk and colleagues and by Dickey and Perrot have corroborated these initial findings [93,94]. Another important point worth mentioning about these studies is the window of opportunity for antibiotic administration. In these studies, when antibiotics were delivered 12 h after phage application, higher bacterial suppression was observed compared to when antibiotics were delivered after 24 h. Others have supported these observations suggesting that there is a specific time window during which subsequent administration of antibiotics results in optimal bactericidal activity [120]. Identifying this time window is critical for future therapeutic applications. The use of other bacteria in combination with phages has shown some remarkable findings. The combination of *Staphylococcal epidermis*, a normal skin microflora with phage saGU1, inhibited the growth of phage-resistant *S. aureus* in vitro [134]. However, this finding cannot be confirmed as no synergistic effect was observed in their in vivo experiment. 

However, with all the foregoing data on the diversity of phage therapy, both in vitro and in vivo, which greatly outweigh any specific concerns, we summarize some important clinical studies that used phages or phage-related *S. aureus* anti-biofilm agents for the treatment of staphylococcal infections over the current time period, as shown in Table 1. In clinical settings, limited knowledge of the usage of phages has been recognized. To our knowledge, there is no phage product currently approved for humans, according to the European Medicines Agency (EMA) or the Food and Drug Administration (FDA) [135]. Nonetheless, phages are mostly utilized for salvage therapy, and the therapeutic evaluation is only based on clinical case series or reports [136]. In fact, there are only 27 clinical trials registered on the clinicaltrials.gov website about bacteriophage therapy since April 2022. Phage therapy (PT) is used in the majority of the cases that are related to biofilms in *S. aureus* infections. These infections are mainly attributed to medical devices [137,138], implants [139,140,141,142,143,144,145,146,147], or grafts [142,148], where biofilms are usually formed by *S. aureus* on these devices. Phage therapy is also included in the management of patients with deep-seated infections such as osteomyelitis [6,149,150,151] (chronic wound/ulcers [152], chronic rhinosinusitis [153], and brain empyema [154]) and chronic prostatitis [155]. However, only one case of healthy children requires exebacase for disseminated MRSA infections [156]. Exebacase is a bacteriophage gene-derived lysin that is used as a single-patient FDA investigational new drug in *S. aureus* infections [157]. Both single or cocktail PT formulations with different dosage regimes are used as therapeutic agents in these cases (Table 1). Phage therapy is also used in patients with only *S. aureus* [6,137,138,139,140,141,142,143,144,146,147,148,150,152,153,154,156] or polymicrobial [142,145,149,150,151,155] detected as causative agents in their clinical specimens. It is also interesting to know that PT is only given intravenously [137,139,146,156] or only given locally to the affected organs/tissues devices or grafts [6,140,141,142,144,145,148,149,151,152,153,154] as well as by multiple routes [138,142,143,147,150,155] The use of PT is also combined with other treatment modalities including antimicrobials alone [6,137,139,142,153,154] and surgical procedures alone [148] or both approaches [138,140,141,143,144,145,146,147,149,151,156]. Interestingly, there are only four cases that used PT alone [150,152,155], with two cases without antibiotics [148,155]. The longest duration of PT is observed in a patient with diabetic foot osteomyelitis, which is seven weeks in total [6]. Several patients had a mild adverse event during PT, that is, transaminitis [138,146,147]. Most of the *S. aureus* biofilm-related infections resolved with PT except in only two patients [142,145], indicating the promising role of PT in human infections. Although narrative in nature and with potential biases, our review could give an insight into the future direction of PT.

The knowledge gained from work performed in this research space will identify opportunities to translate phage therapy into practice for *S. aureus* infections, as well as to address knowledge gaps or issues that need to be addressed. As highlighted, phage offers an extremely promising therapeutic alternative to combat staphylococcal infection found in various human diseases, including septicemia, pneumonia-like infection, venous leg ulcers, etc. [158]. Phages are characterized by their effective eradication capacity against specific multidrug-resistant bacteria and associated low costs when compared to antibiotics. Mainstream phage therapy is still evolving, and the knowledge gained from each study is critically needed to understand the factors that influence their safety and efficacy in medicine and other sciences. Specific to *S. aureus* infections, it is important to highlight that relatively few phages would be needed and combined with currently available antibiotics to have an effective outcome. Furthermore, different phages can be combined into unique cocktails and administered via different routes, i.e., topically, orally, or both, to facilitate effective penetration into the system/tissue [99].

However, several challenges have also been identified. For example, lysogenic phages are commonly considered unsuitable for treatment due to their high potential for horizontal gene transfer; however, some lytic phages show no positive value as antimicrobial agents [159]. Performing whole genome sequencing allows one to avoid the application of phages that carry toxic genes [160]. Additionally, phages can interact with the immune system and trigger a response that may reduce the efficiency of phage therapy [161]. It has also been shown that the spleen can inactivate phages in blood circulation [162], and allergic reactions can narrow the potential use of phages [163]. Nonetheless, little or no impact caused by the immune response on the potential killing of bacteria by phages is expected as bacterial lysis occurs before a specific antibody is formed. In addition, there is no increase generation of pro-inflammatory cytokines or reactive oxygen species following the administration of phages, thus, limiting tissue damage [164]. Other challenges include the fact that bacterial cells are able to develop resistance mechanisms against phages (e.g., modification of surface receptors via mutations), which can be disseminated to other bacterial cells as a result of selective pressures generated by phages during treatment periods [165]. However, the amount of inoculum of phages during its administration with or without antibiotics may play an important role in minimizing the risk of resistance [166]. Phages that are highly bactericidal will be able to kill the bacteria faster than they can replicate; hence, a higher inoculum of phages may be considered in the formulation of phage cocktails. It is also important to note that staphylococci include numerous clones. Therefore, it may be necessary to identify phage(s) based on these clone types and also the type of specific strains. The bactericidal activity of phages towards *S. aureus* isolates has been shown to be significantly associated with the type of the *S. aureus* clonal complex (CC). Phage V1SA20 is only active against CC80 strains, while all phages exhibit poor activity against CC7, CC59, CC239, and CC398 strains [167]. However, the host immune response and the nature of the infection may influence the efficacy of phage therapy; even strain-specific phage is used [4].

Phage therapy (PT) should also be carefully assessed in patients with chronic infections caused by polymicrobial agents. For example, anti-*S. aureus* PT failed to completely cure chronic polymicrobial biofilm infection of a bone allograft in a sarcoma patient. The authors explained the possibility of incomplete coverage of anti-*S. aureus* PT against non-staphylococci bacteria [151]. In addition, a special precaution should be implemented when PT is used when treating infections in patients with cancers. Following the introduction of bacteriophages (T4 and M13) into prostate cancer cell lines (PC-3), overexpression of integrins was observed, which may be beneficial to prostate cancer cases [168]. However, it may be detrimental to other types of cancers, such as ovarian and breast cancers. Overexpression of selected integrins (ITGAV and ITGB3) would promote the survivability of these cancer cells and their proliferation [169]. Although there is a lack of phage receptors in mammalian cells, Bichet et al. [170] demonstrated the uptake of phages by several cell lines through micropinocytosis in vitro [170]. The uptake of phages and their internalization process across the cell lines are very heterogenous, and the authors concluded that the type of cells and phages have no roles in their specificity. Interestingly, the viability of these phages was lost due to the inactivation process [170]. This finding could further compromise the accessibility of phages to the site of infection in the tissues or organs during phage therapy. Modifications in the shape of phages may reduce the sequestration issues related to cellular uptake during phage therapy. It has been shown that the elongated-shaped phages have lower uptake rates by the cells compared with the disc-shaped phages [171]. Tolerability and safety for patients are paramount but are affected by the solution formulations phages are stored in. Formulations need to be specifically designed in order to maintain phage stability in storage, which, when poorly formulated, would impact effectiveness during treatment [172]. On a similar note, an appropriate therapeutic regime (dosage, dosing interval, and timing) is also pivotal in ensuring the efficacy of phage therapy, as clearly explained by [4].

Work currently being performed is also identifying phage-associated treatment options. For example, phages can produce specific enzymes (e.g., endolysin), which are involved in the rapid degradation and destruction of the bacterial cell wall. The promising findings on phage-encoded endolysins from in vitro studies have been extensively reviewed, and their synergy action with antibiotics could give insight into new therapeutic options [173]. This enzyme has shown bactericidal activity when added exogenously and is a potential alternative to using live or engineered phages [174]. Furthermore, the possibility of bacteria developing resistance to endolysin activity is low since endolysins target unique and highly conserved peptidoglycan bonds [175]. When considered as a collective, the benefits of phage therapy appear to outweigh any identified concerns greatly; however, more research is necessary to address these and facilitate mainstream acceptance of this therapy. 

## 3. Conclusions

This review summarises the essential aspects of antibiotic resistance observed in *S. aureus* that must be addressed and understood in order to facilitate the development of phage therapy into standard clinical practice. Overall, several points emerge from this review. Firstly, positive interactions that are observed between phages and antibiotics provide a strong rationale that phage/antibiotic combinations can be successfully used against many multidrug-resistant bacteria [121,176]. Secondly, the effectiveness of such combined therapy witnessed in vitro would have to be corroborated in relevant in vivo systems to rationale clinical trials. Thirdly, research is desperately needed to deduce how phage formulations work, considering they each exhibit their unique pharmacodynamic properties [94]. Finally, the chronological administration of phages and antibiotics appears to be the most promising approach to combat infectious biofilms. However, since the vast majority of biofilms evolve from mono-species into polymicrobial biofilms, successful treatment of these in the future may need to be comprised of multiple-species phage cocktails targeting different bacterial receptors [93,133]. Nevertheless, the encouraging results obtained are a great effort to continue experimentation with phage/antibiotic combinations and are likely to reap the rewards of an alternative treatment to antibiotics in the near future.

## Figures and Tables

**Table 1:** Overview of the application of phage and phage-related therapy against *Staphylococcus aureus* biofilm-related infections in humans between 2018 and 2022.

Year	Clinical Condition	Bacterial Pathogen	Phage Source	Phage Preparation	Phage Dose (PFU/mL)	Number of Doses	Route of Administration	Combined Therapy	Treatment Duration	Clinical Outcome	Adverse Events	Reference
2018	Diabetic foot osteomyelitis	MSSA	Eliava Institute	Single Phage	NR	Multi-dose	Local	Levofloxacin (stopped on Day 7)	7w	Resolved	NR	[6]
2019	Device-related infection	MSSA	AmpliPhi Biosciences	Phage Cocktail	3.0 × 10^9^	Multi-dose	Intravenous	Cefazolin and minocycline	4w	Resolved	No	[137]
2019	Osteomyelitis	S. aureus *	Queen Astrid Military Hospital	Phage cocktail	1.0 × 10^7^	Multi-dose	Local	Amoxicillin, vancomycin, rifampicin, moxifloxacin, colistin, fosfomycin and clindamycin, and surgery	7–10 d	Resolved	No	[149]
2019	Chronic wound	*S. aureus*	Banaras Hindu University	Phage cocktail	1.0 × 10^9^	Multi-dose	Local	No	13 d	Resolved	No	[152]
2019	Device-related infection (prosthetic valve endocarditis)	MSSA	AmpliPhi Biosciences	Phage cocktail	1.0 × 10^9^	Multi-dose	Intravenous	Flucloxacillin, ciprofloxacin and rifampicin	2w	Resolved	No	[139]
2020	Cranial empyema	MSSA	Pherecydes Pharma	Phage Cocktail	NR	Single-dose	Local	Dalbavancin	NA	Resolved	No	[154]
2020	Device-related infection (knee prosthetic)	MSSA	Pherecydes Pharma	Phage Cocktail	1.0 × 10^9^	Single-dose	Local	Daptomycin, cloxacillin, levofloxacin and rifampicin, and surgery	NA	Resolved	NR	[141]
2020	Device-related infection (knee prosthetic)	MRSA	Adaptive Phage Therapeutics	Single Phage	5.4 × 10^9^	Multi-dose	Dual routes (local and intravenous)	Daptomycin and surgery	3 d	Resolved	Reversible transaminitis	[138]
2020	Osteomyelitis	MSSA	LTD Eliava Biopreparations	Phage Cocktail	1.0 × 10^7^	Multi-dose	Dual routes (local and oral)	No	5w	Resolved	No	[150]
	Osteomyelitis	MSSA	LTD Eliava Biopreparations	Phage Cocktail	1.0 × 10^7^	Multi-dose	Dual routes (local and oral)	No	5w	Resolved	No	
	Diabetic foot osteomyelitis	S. aureus *	LTD Eliava Biopreparations	Phage Cocktail	1.0 × 10^7^	Multi-dose	Dual routes (local and oral)	No	6w	Resolved	No	
2020	Device-related infection (knee prosthetic)	MSSA	Pherecydes Pharma	Phage Cocktail	1.0 × 10^9^	Single-dose	Local	Daptomycin, levofloxacin, ofloxacin and doxycycline, and surgery	NA	Resolved	NR	[140]
	Device-related infection (knee prosthetic)	MSSA	Pherecydes Pharma	Phage Cocktail	1.0 × 10^10^	Single-dose	Local	Daptomycin, ceftazidime, ciprofloxacin and rifampin, and surgery	NA	Improved clinically	No	
	Device-related infection (knee prosthetic)	MSSA	Pherecydes Pharma	Phage Cocktail	1.0 × 10^9^	Single-dose	Local	Daptomycin, cefepime, rifampin and levofloxacin, and surgery	NA	Improved clinically	NR	
2020	Device-related infection	S. aureus *	Gabrichevsky Institute	Phage cocktail	1.0 × 10^8^	Multi-dose	Dual routes (local and oral)	Cefepime, daptomycin, linezolid and tobramycin	NA	Resolved but died of other complications	No	[142]
	Device-related infection	*S. aureus*	Gabrichevsky Institute	Single Phage	1.0 × 10^9^	Multi-dose	Dual routes (local and oral)	Rifampicin, flucloxacillin	2 d	Resolved	No	
	Device-related infection	*S. aureus*	Gabrichevsky Institute	Single Phage	1.0 × 10^9^	Multi-dose	Dual routes (local and oral)	Daptomycin	12 d	Resolved but died of other complications	No	
	Device-related infection	*S. aureus*	Gabrichevsky Institute	Phage cocktail	1.0 × 10^9^	Multi-dose	Multiple (local, inhaled, and oral)	Daptomycin	8 d	Not resolved	No	
	Device-related infection	*S. aureus*	Gabrichevsky Institute	Single Phage	4.0 × 10^10^	Single-dose	Local	Sultamicillin	NA	Resolved	No	
2021	Chronic bacterial prostatitis	MRSA *	Eliava Institute	Phage Cocktail	1.0 × 10^5^–1.0 × 10^7^	Multi-dose	Multiple (oral, intra-rectal, and intra-urethral)	No	NA	Resolved	No	[155]
2021	Osteomyelitis	MSSA *	Queen Astrid Military Hospital	Phage Cocktail	1.0 × 10^7^	Multi-dose	Local	Clindamycin, rifampin and ciprofloxacin, and surgery	2w	Clinical improvement	NR	[151]
2021	Device-related infection (knee prosthetic)	MSSA	Adaptive PT	Single Phage	2.9 × 10^10^	Multi-dose	Dual routes (local and intravenous)	Cefazolin and surgery	6w	Resolved	No	[143]
2022	Device-related infection	*S. aureus*	Sanubiom GmbH, Fritzens, Austria	Phage Cocktail	1.0 × 10^7^	Single-dose	Local	Piperacillin/tazobactam and surgery	NA	Resolved	NR	[144]
2022	Chronic rhinosinusitis	MRSA	Adaptive Phage Therapeutics	Single Phage	1.0 × 10^9^–1.0 × 10^10^	Multi-dose	Local	Oritavancin	3w	Resolved	NR	[153]
2022	Device-related infection	MSSA *	Sanubiom GmbH, Fritzens, Austria, Phage 24.com	Phage Cocktail	1.0 × 10^7^	Single-dose	Local	Piperacillin/tazobactam and surgery	NA	Not resolved	NR	[145]
2022	Device-related infection	MSSA	Phage24.com, Austria	Phage Cocktail	NA	Single-dose	Local (endovascular grafts coated with phage)	Surgery	NA	Resolved	NR	[148]
2022	Device-related infection	MRSA	NA	Single Phage	1.0 × 10^9^–1.0 × 10^10^; 2.0 × 10^8^	Multi-dose	Intravenous	Daptomycin and ceftaroline and surgery	3 d	Resolved	Reversible transaminitis	[146]
2022	Device-related infection	MRSA	Adaptive PT (Gaithersburg, MD, USA)	Single Phage	1.2 × 10^9^; 2.4 × 10^7^–2.4 × 10^8^; 3.0 × 10^8^–4.0 × 10^8^	Multi-dose	Dual routes (local and intravenous)	Daptomycin and Bactrim and surgery	3 d	Resolved	Reversible transaminitis	[147]
2022	Disseminated *S. aureus* infections: meningitis, retropharyngeal abscess, cranial empyema, and endocarditis	MRSA	ContraFect Corp	exebacase	3 mg	Single-dose	Intravenous	Vancomycin, linezolid, daptomycin and ceftaroline, and surgery	NA	Resolved	NR	[156]

MSSA—Methicillin-sensitive *Staphylococcus aureus*; MRSA—Methicillin-resistant *Staphylococcus aureus*; NA—Not applicable; NR—Not reported; PFU—Plaque-forming unit; *—More than one bacterium/case series (polymicrobial); PT– Phage therapy; d—day; w—week; Note: Device-related infection includes implants, grafts, and prosthetics.

## Data Availability

All the data related to this review are included in the manuscript.
